# Effects of 14-weeks betaine supplementation on pro-inflammatory cytokines and hematology status in professional youth soccer players during a competition season: a double blind, randomized, placebo-controlled trial

**DOI:** 10.1186/s12970-021-00441-5

**Published:** 2021-06-05

**Authors:** Hadi Nobari, Jason M. Cholewa, Jorge Pérez-Gómez, Alfonso Castillo-Rodríguez

**Affiliations:** 1 Department of Exercise Physiology, Faculty of Sport Sciences, University of Isfahan, Isfahan, 81746-7344 Iran; 2 Department of Exercise Physiology, College of Health 587 Sciences, University of Lynchburg, Lynchburg, 24501 VA USA; 3 HEME Research Group, Faculty of Sport Sciences, University of Extremadura, Cáceres, 10003 Spain; 4 Department of Physical Education and Sports, University of Granada, Granada, 18010 Spain

**Keywords:** Nonfunctional over-reaching, Youth sports, Immune system, Football, IL-1β

## Abstract

**Objective:**

Systemic elevations in pro-inflammatory cytokines are a marker of non-functional over reaching, and betaine has been shown to reduce the secretion of pro-inflammatory cytokines in vitro. The aim of this study was to investigate the effects of betaine supplementation on tumor necrosis factor alpha (TNF-α), interleukins-1 beta (IL-1β), − 6 (IL-6) and the complete blood cell (CBC) count in professional youth soccer players during a competitive season.

**Methods:**

Twenty-nine soccer players (age, 15.5 ± 0.3 years) were randomly divided into two groups based on playing position: betaine group (BG, *n* = 14, 2 g/day) or placebo group (PG, *n* = 15). During the 14-week period, training load was matched and well-being indicators were monitored daily. The aforementioned cytokines and CBC were assessed at pre- (P1), mid- (P2), and post- (P3) season.

**Results:**

Significant (*p* < 0.05) group x time interactions were found for TNF-α, IL-1β, and IL-6. These variables were lower in the BG at P2 and P3 compared to P1, while IL-1β was greater in the PG at P3 compared to P1 (*p* = 0.033). The CBC count analysis showed there was significant group by time interactions for white blood cells (WBC), red blood cells (RBC), hemoglobin (Hb), and mean corpuscular hemoglobin concentration (MCHC). WBC demonstrated increases at P3 compared to P2 in PG (*p* = 0.034); RBC was less at P3 compared to P1 in BG (*p* = 0.020); Hb was greater at P2 compared to P1, whilst it was less at P3 compared to P3 for both groups. MCHC was greater at P3 and P2 compared to P1 in BG, whereas MCHC was significantly lower at P3 compared to P2 in the PG (*p* = 0.003).

**Conclusion:**

The results confirmed that 14 weeks of betaine supplementation prevented an increase in pro-inflammatory cytokines and WBC counts. It seems that betaine supplementation may be a useful nutritional strategy to regulate the immune response during a fatiguing soccer season.

## Introduction

Soccer is the most popular sport in the world, performed by men and women of varying ages and skills [1]. Youth participation in soccer has increased world-wide in the past two decades, and many youth players aspire to become professional soccer players [2]. Soccer is a high effort intermittent sport with highly varied metabolic and movement patterns. Youth soccer players cover an average of 6 to 8 km per game, with sprints occurring approximately every 90 s, comprising 1–11% of total distance, and approximately 81 accelerations (<− 1 m.s^− 2^) and 82 decelerations (> − 1 m.s^− 2^) performed per game [3–7]. The average heart rate during a professional youth game has been reported between 82 and 87% HR_max_ [8, 9] and lactate levels between 3 to 8 mmol/L [10].

Monitoring training load, recovery, and changes in psychological status provides useful information for coaches to manage variations in intensity and individualize training in order to reduce the risk of injury and the development of non-functional overreaching syndrome (NFOR) [11, 12]. When the volume of training/competition increases in conjunction with insufficient recovery, players may enter into an NFOR marked by decrements in performance and greater incidences of injury and illness due to cumulative fatigue during the season [13]. On the other hand, regular monitoring of endocrine hormones may be used as biomarkers of physiological stress which may influence recovery and performance throughout the season. Particularly, changes in growth hormone, cortisol, and the testosterone/cortisol ratio, in addition to biomarkers of the immune system (i.e.: interleukins), have all been reported as reliable biomarkers that may illustrate NFOR in athletes [13, 14]. In some studies as well, to monitor the training load of rating of perceived exertion [7, 15] and Hooper index (i.e.: perception of fatigue, stress, delayed onset muscle soreness, or sleep quality) has also been used to identify the well-being status of players and their variation across critical periods of the season, which can be other options for identifying NFOR [16, 17].

Due to intense training, competitions, and match-related stress, soccer players experience homeostatic, biochemical, and hematological changes following a soccer match and across a competitive season [18]. Large effect sizes have been reported for the inflammatory and immunological response immediately and up to 72 h following a soccer match. This response includes an elevation in tumor necrosis factor alpha (TNF)-α and pro-inflammatory interleukins (IL) [19] that are produced by skeletal muscle, T-cells, and natural killer cells [20]. The best-studied cytokine that is related to exercise is interleukin-6 (IL-6), which can act as both a pro- and anti-inflammatory cytokine [21], and it is sensitive to both the intensity and volume of exercise in addition to metabolic stress and muscular injury [22, 23]. TNF-α is part of the nuclear factor kappa b (NFκB) pathway, and produced predominantly by macrophages in response to injured skeletal muscle tissue [24]. In addition to inflammatory cytokines, hemoglobin (Hb), hematocrit (Hct), and mean corpuscular hemoglobin concentration (MCHC) are diminished following a soccer match [25], and a large increase in circulating leukocytes and specific monocytes, macrophages and lymphocytes occurs [26] as these cells rapidly migrate to injured muscle tissue.

Regular bloodwork, including measuring complete blood cell count (CBC) with differential and inflammatory cytokines, may be used as indicators of training stress associated with NFOR [27]. Walker et al. reported that IL-6 significantly increased over the duration of a soccer season, and the greatest elevations in IL-6 were observed during the highest states of physiological demand occurring at the end of the season [28]. This pro-inflammatory state was suggested to disrupt the hypothalamic-pituitary axis leading to greater circulating cortisol concentrations, and it is an indicator of NFOR [28, 29]. Red blood cells (RBC) and platelets (Pts) are also sensitive to systematic inflammatory changes, and may be used to monitor NFOR [30]. Huggins et al. reported a significant decrease in Hct and Hb from pre-season to the fourth week that remained depressed throughout the season [31]. Huggins et al. also reported a regression in anemia indicators, such as mean corpuscular volume (MCV), mean corpuscular hemoglobin (MCH), and red cell distribution width (RDW) at the end of the season, in addition to an increase in MCHC levels between pre-season and the fourth week [31].

Dietary supplements have become popular in the sports community over the past few decades to support adaptation and manage fatigue, however, research suggests that only a small group of supplements are safe and effective for athletes [32, 33]. Betaine is a zwitterionic quaternary ammonium compound, a byproduct isolated from molasses during sugar beet refinement, and is naturally occurring in spinach, whole grains, and seafood. The daily average betaine intake is approximately 100–400 mg [34], and several studies suggest that 2.0–2.5 g of betaine supplementation per day may be consider as an ergogenic aid [35, 36]. Although the mechanisms by which betaine may be ergogenic are not fully understood, chronic betaine supplementation may enhance recovery between training sessions by protecting against protein denaturation and promoting the secretion of insulin growth factor-1 and protein kinase B phosphorylation [37].

Inverse correlations between betaine ingestion and markers of inflammation (C reactive protein and TNF-α) were first reported in 2008 [38], and since several studies have demonstrated a causal relationship [39]. In regards to exercise recovery, IL-1β accumulation leads to intramuscular inflammation following eccentric exercise [40]. Betaine has been shown to inhibit NFκB activity [41], potentially dampening the inflammatory response to exercise by lowering the production and secretion of IL-1β, IL-6 and TNF-α [42]. We recently reported that betaine supplementation prevented a reduction in the testosterone to cortisol ratio associated with the demands of a competitive season in professional youth soccer players [13]. Currently there is limited research examining the influence of betaine supplementation on markers of inflammation or blood cell parameters. Given the positive effects of betaine supplementation on hormones associated with NFOR, and in vitro evidence demonstrating an anti-inflammatory effect of betaine, betaine supplementation may also offset the development of NFOR by ameliorating the inflammatory response to chronic, strenuous exercise, such as that required by professional soccer players.

This study is an extension of an ongoing study exploring the effects of betaine supplementation on markers of NFOR in professional youth soccer players [13]. The purpose of this study was to investigate the effect of betaine supplementation on pro-inflammatory cytokines and CBC in professional youth soccer players during a 14-week competitive season. We hypothesize that betaine supplementation will reduce markers of inflammation and indicators of anemia.

## Material and method

### Selection of study groups

Professional youth soccer players (*n* = 29), from the Foolad Mobarakeh Sepahan Sport Club competing in the Iranian Youth Premier League, participated in this study. Demographics, inclusion, and exclusion criteria associated with this sample has been previously published [13]. In brief, subjects were required to attend all training sessions, not consume any dietary supplements during the study period, not perform any non-team training, and have no records of sensitivity to dietary supplements in the team medical records. Subjects were matched according to position prior to randomization into either a betaine (BG, *n* = 14) or placebo (PG, *n* = 15) group (Table 1). Players, as well as their parents, signed a consent letter to participate. The Ethics Committee of the University of Isfahan approved the study (IR.UI.REC.1398.102), and the recommendations of Human Ethics in Research were followed by the Helsinki Declaration (2013).

**Table 1:** Descriptive characteristics of the soccer players (*n* = 29) U16 by groups

Variables	Groups	***Mean ± SD***	Confidence Interval 95%
Height (cm)	*BG*	172.1 ± 2.3	[170.8 to 173.5]
	*PG*	174.2 ± 4	[171.9 to 176.4]
Body mass (kg)	*BG*	59.2 ± 4.8	[56.4 to 61.9]
	*PG*	65.8 ± 7.0	[61.9 to 69.6]
BMI (kg.m^2^)	*BG*	18.8 ± 4.7	[16.1 to 21.6]
	*PG*	21.7 ± 1.8	[20.7 to 22.6]
VO_2max_ (ml.kg^− 1^.min^− 1^)	*BG*	48.7 ± 2.2	[47.4 to 50.0]
	*PG*	47.5 ± 2.6	[46.1 to 48.9]
PHV (years)	*BG*	13.6 ± 0.2	[13.3 to 13.7]
	*PG*	13.4 ± 0.5	[13.1 to 13.6]
Maturity Offsets (years)	*BG*	1.8 ± 0.2	[1.6 to 2.0]
	*PG*	2.1 ± 0.4	[1.8 to 2.3]
Age (years)	*BG*	15.4 ± 0.3	[15.3 to 15.5]
	*PG*	15.5 ± 0.2	[15.3 to 15.6]
Body Fat (%)	*BG*	8.7 ± 2.7	[7.1 to 10.2]
	*PG*	9.2 ± 3.5	[7.3 to 11.1]
LBM (kg)	*BG*	54.0 ± 4.6	[51.4 to 56.7]
	*PG*	59.6 ± 5.4	[56.7 to 62.6]

*SD* Standard deviation, *BG* Betaine group, *PG* Placebo group, *BMI* Body mass index, *VO*_*2max*_, *PHV* Peak height velocity; *LBM* Lean body mass

### Experimental approach to the problem

The present study was an independent group, pre-test, mid-test, and post-test of an experimental design. Studies in humans show that betaine is rapidly absorbed from the intestine and reach peak serum concentrations within 1–2 h [43]. Based on this reason, subjects consumed one capsule, betaine or placebo, twice daily (2 g/day), with 300 ml water approximately, 2 h prior to training and one-hour post-training, or with lunch and dinner on non-training days. Anthropometric measurements, body composition, and blood tests were conducted before week 1 (P1), seventh week (P2) and after 14 weeks (P3) of the intervention, and were performed 48 h apart from the last training session. Subjects recorded their nutrition for three full days and delivered it to the researchers at each testing time-point. All players participated in the same standardized training sessions during the study (i.e., four training sessions and one match per week). In order to match the load of the weekly workouts, players who did not participate in competitions performed post-match, small side games, individual training, or friendly competition. The subjects presented individual wellness questionnaires before the start of each training session, and reported internal training load via rating of perceived exertion (RPE) 30 min after the end of each training session.

### Procedures

#### Blood analysis

Subjects reported to the Alzahra Hospital’s lab for blood sampling following a 12 h fast, and at least 48 h following the last training session. To account for circadian rhythms, 10 cc of blood was collected from the antecubital vein at 8–9 a.m. Samples were immediately centrifuged, the serum was separated, and then used to measure CBC, IL-1β, IL-6, and TNF-α on the same day.

##### Analyzing hematology levels

The CBC count consisted of neutrophils, lymphocytes, and the combination of eosinophils, basophils, and monocytes (MIX). To obtain the ratio of neutrophils to lymphocytes (NLR) these two variables were divided together. Total RBCs were measured in addition to MCV, MCH, RDW. The Hb, Hct, and Pts determinations were performed on plasma anticoagulated with EDTA using a fully-automated hematology analyzer (Sysmex kx-21 N Kobe, JAPAN) according to the manufacturer’s recommended protocols.

##### Analyzing cytokine levels

Inflammatory markers were measured using enzyme-like immune-sorbent assay (ELISA reader, awareness technology, USA). All variables were measured with kits obtained from Diaclone, Besançon, France. Serum concentrations were performed for IL-1β with the sensitivity of the kit 6.5 pg/mL. The sensitivity of IL-6 was 2 pg/mL and average inter- and intra-assay coefficients of variability (CVs) were 7.7 and 3.6%, respectively. TNF-α had a sensitivity 8 pg/mL and average inter- and intra-assay CVs were 10.9 and 3.2%, respectively. All analyses were performed in duplicates.

#### Anthropometric and body composition

Detailed methods regarding the measurement of anthropometrics and body composition have been previously reported for this sample [13]. In brief, standing height with a stadiometer (Seca 213, Germany), weight with a balance scale (Seca 813, UK), and body composition with 7 site skin folds (Lafayette Instrument Company, Lafayette, IN, USA) and Brozek’s formula [44]. To determine the maturity offset and age at peak height velocity of the subjects the following formula was used [45]: Maturity offset = − 9.236 + 0.0002708 (leg length × sitting height) − 0.001663 (age × leg length) + 0.007216 (age × sitting height) + 0.02292 (Weight by Height ratio).

#### Aerobic power test

An Intermittent Fitness Test 30–15 (30-15_IFT_) was used to estimate the maximal oxygen uptake (VO_2max_) and the readiness level of the subjects. This test was performed after the P1 evaluations and before the start of the competition season (i.e., week 1). All subjects performed 10 min of standard warm-up, include jogging, dynamic stretching, ABC run drills (e.g., high knee, A-Skip, B-Skip, carioca, etc.), and submaximal short runs (2–3 rep), under the guidance of the team fitness coach.

After the warm-up, subjects were placed in four-person groups. The 30-15_IFT_ includes a 40-m shuttle with 30 s of activity and 15 s of passive recovery. The first stage was 30 s and initial speed started with 8 km/h^− 1^ and increased by 0.5 km/h^− 1^ every 45 s [46]. This test was terminated when subjects could not continue or for three consecutive shuttles could not maintain the appropriate pace. The following formula was used to estimate the VO_2max_ [46]**:** VO_2max_ (ml.kg^− 1^.min^− 1^) = 28.3 – (2.15 × 1) – (0.741 × 16 years) – (0.0357 x Weight) + (0.0586 × 16 years x VIFT) + (1.03 x VIFT). Where VIFT is the final running speed. The test-retest of this assessment at the intra-class correlation coefficient (ICC) of this test was 0.91. This test has shown high validity (ICC = 0.96) in soccer players [47].

#### Control of food intake

Detailed methods regarding the dietary standards and measurement of energy and macronutrient intake have been previously described and published for this sample [13]. In brief, subjects were provided with nutrition recommendations of Iranian native foods that provided energy equal to 1.55 times the individual subjects basal metabolic rate by a nutritionist. Subjects were required to consume the same foods and record these intakes 72 h prior to each blood sample, and total calorie intake was measured with Nutrition 4 version 3.5.2 software [48].

#### Monitoring internal training loads

Internal training loads in soccer players were assessed with a 10-piont session RPE and have been described in detail and previously reported for this sample [13]. Training load monitoring was performed 30 min after each training session. This is the standardized approximate time according to previous studies [15, 49–51].

#### Wellness monitoring

Hopper index was used to assess fatigue, recovery, soreness, quality of sleep, and health status of soccer players in each session [52–54]. Detailed methods and results have been previously described and published for this sample in relation to the Hooper index [13]. Players had an average of 5 training sessions per week for 14 weeks. Immediately after attending the training, they answered Hooper’s 7-score questionnaire with the above variables mentioned [16, 17].

### Statistical analysis

Descriptive statistics are reported as mean ± standard deviation. Shapiro-Wilk test and Levene’s test were used to check the normality and homogeneity of variables of data, respectively. All variables were assessed with a mixed factorial 2 × 2 analysis of covariance (ANCOVA) with repeated measures. The pre-season value was used as the covariate, time (mid-season and post-season) the within subject factor, and BG and PG the between subject factor. When a significant time x group interaction was found a one-way repeated-measures analysis of variance (ANOVA) was conducted for each group separately with the Bonferroni correction. If the results of the one-way ANOVA were similar for each group, then the percent changes were computed. Hedge’s g effect size (95% confidence interval) was computed to define the magnitude of comparisons pre- and post-season for both groups by separately. Threshold’s include: trivial (< 0.2), small (≥ 0.2), moderate (≥ 0.5) and large (≥ 0.8). All analyses were conducted with SPSS 22.0 (IBM) and the significant level was set at *p* < 0.05. also, Excel was used for the training workload and hopper data and the charts were drawn with GraphPad Prism 8.0.1.

## Results

**Fig. 1:**
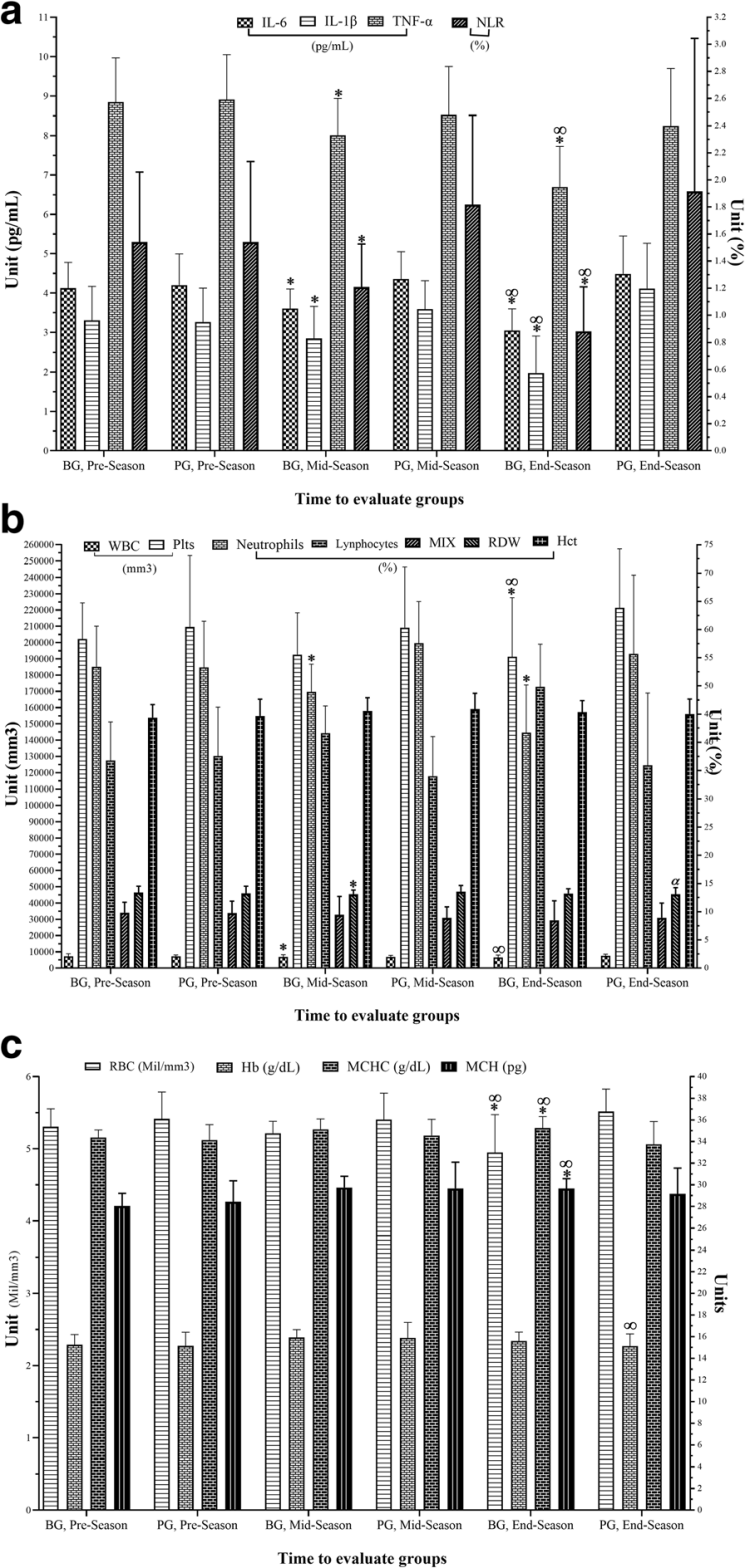
Change in all blood sampling variables assessment for each group and assessment stage. **a** Indicates changes in pro-inflammatory cytokines, and NLR; **b** Indicates changes in types of leukocytes, Plts, and Hct; **c** Indicates changes to the rest of RBC variables. * significant difference compared to P1 with the superiority of the BG (*p* < 0.05); ^#^ significant difference compared to P1 with the superiority of the PG (*p* < 0.05); ∞ significant difference compared to P2 with the superiority of the BG (*p* < 0.05); ^α^ significant difference compared to P2 with the superiority of the PG (*p* < 0.05). BG: Betaine Group; PG: Placebo Group; IL-6: Interleukin-6; IL-1β: Interleukin-1 beta; TNF-α: Tumor necrosis factor-alpha; WBC: White blood cells; MIX: Plural of eosinophils, basophils, and monocytes; NLR: Neutrophils to lymphocytes ratio; RBC: Red blood cells; MCH: Mean corpuscular hemoglobin; MCHC: Mean corpuscular hemoglobin concentration; RDW: Red cell distribution width; Hb: Hemoglobin; Hct: Hematocrit; Plts: Platelets; P1: Pre-season; P2: Mid-season; P3: Post-season

There were no significant main effects of time for changes in IL-6 (*p* = 0.88, *F* = 0.022, η_p_^2^ = 0.001) and TNF-α (*p* = 0.84, *F* = 0.04, η_p_^2^ = 0.002), however, there were significant group by time interactions (*p* = 0.008, *F* = 8.11, η_p_^2^ = 0.24) and (*p* = 0.038, *F* = 4.78, η_p_^2^ = 0.16), respectively. Post hoc analysis revealed the IL-6 and TNF-α significantly decreased from P1 to P2 and P3, as well as from P2 to P3 in the BG, whereas there were no significant differences between P1, P2, or P3 in the PG. There were no significant (*p* = 0.69, *F* = 0.16, η_p_^2^ = 0.01) main effects of time, however, there was a group by time interaction (*p* ≤ 0.001, *F* = 16.02, η_p_^2^ = 0.38) for changes in IL-1β. The IL-1β at P3 and P2 were significantly lower than P1, as well as significantly lower than from P2 to P3 only in BG. In PG the IL-1β was significantly greater at P3 compared to P1 (*p* = 0.033) (Table 2). Figure 1a shows the comparison of the mean difference between-group results of the one-way ANOVA at the three assessments stages. For the BG it was observed that the level of IL-6 (*p* < 0.05), IL-1β and TNF-α (*p* ≤ 0.001) were significantly lower for P2 and P3.

**Table 2:** Changes in cytokines levels during pre- mid- and post-season. Mean (M) ± standard deviation (SD)

Variables	Groups	Pre-Season	Mid-Season	Post-Season	Pre-Post Season		95% CI Hedge’s g	
		M ± SD	M ± SD	M ± SD	% Change	Hedge’s g	Lower	Upper
IL-6 (pg/mL)	*BG*	4.13 ± 0.65	3.60 ± 0.50^€^	3.05 ± 0.55^#*^	−26.1	−1.69 L	− 2.56	−0.83
	*PG*	4.20 ± 0.79	4.36 ± 0.69	4.49 ± 0.96	6.8	0.31 S	− 0.41	1.03
IL-1β (pg/mL)	*BG*	3.16 ± 0.68	2.69 ± 0.59^€^	1.79 ± 0.63^#*^	−40.2	−1.40 L	−2.23	−0.58
	*PG*	3.26 ± 0.86	3.59 ± 0.72	4.12 ± 1.15^*^	26.1	0.79 M	0.05	1.54
TNF-α (pg/mL)	*BG*	8.85 ± 1.12	8.01 ± 0.92^€^	6.70 ± 1.03^#*^	−24.3	−1.89 L	−2.79	−1.00
	*PG*	8.91 ± 1.14	8.52 ± 1.23	8.24 ± 1.45	−7.5	−0.48 M	−1.21	0.24

*BG* Betaine Group, *PG* Placebo Group, *IL-6* Interleukin-6, *IL-1β* Interleukin-1 beta, *TNF-α* Tumor necrosis factor-alpha, *P1* Pre-season, *P2* Mid-season, *P3* Post-season, *T* Trivial, *S* Small, *M* Moderate, *L* Large

^€^ significant difference compared to P1-P2 (*p* < 0.05);

^#^ significant difference compared to P2-P3 (*p* < 0.05);

^*^ significant difference compared to P1-P3 (*p* < 0.05)

There were no significant (*p* = 0.36, *F* = 0.86, η_p_^2^ = 0.03) main effects of time, however, there was a group by time interaction (*p* = 0.002, *F* = 11.49, η_p_^2^ = 0.31) for changes in WBC. This variable demonstrated a significant increase at P3 compared to P2 in PG (*p* = 0.034). There were no significant main effects of time for changes in neutrophils (*p* = 0.60, *F* = 0.28, η_p_^2^ = 0.01), lymphocytes (*p* = 0.69, *F* = 0.17, η_p_^2^ = 0.01), NLR (*p* = 0.76, *F* = 0.10, η_p_^2^ = 0.004) and MIX (*p* = 0.09, *F* = 3.02, η_p_^2^ = 0.10) or group by time interaction (*p* = 0.22, *F* = 1.56, η_p_^2^ = 0.06), (*p* = 0.14, *F* = 2.38, η_p_^2^ = 0.08), (*p* = 0.23, *F* = 1.55, η_p_^2^ = 0.06) and (*p* = 0.44, *F* = 0.61, η_p_^2^ = 0.02), respectively.

There were no significant (*p* = 0.71, *F* = 0.14, η_p_^2^ = 0.01) main effects of time, however, there was a group by time interaction (*p* = 0.02, *F* = 6.22, η_p_^2^ = 0.19) for changes in RBC. This variable was significantly lower at P3 compared to P1 in BG (*p* = 0.020). There was no significant main effect of time for MCV (*p* = 0.83, *F* = 0.05 η_p_^2^ = 0.002) or group by time interactions *(p* = 0.64, *F* = 0.23, η_p_^2^ = 0.01). There were no significant (*p* = 0.50, *F* = 0.46, η_p_^2^ = 0.02) main effects of time, however, there was a group by time interaction (*p* = 0.03, *F* = 5.36, η_p_^2^ = 0.17) for changes in MCH. Post hoc analysis showed the MCH were significantly greater at P3 and P2 compared to P1 for both groups, but MCH was significantly less at P3 compared to P2 in the PG (*p* = 0.003). For MCHC there was significant main effect of time (*p* = 0.04, *F* = 4.79, η_p_^2^ = 0.16) and significant group by time interactions *(p* = 0.001, *F* = 14.73, η_p_^2^ = 0.36). Post hoc analysis showed the MCHC were significantly greater at P3 and P2 compared to P1 in BG, whereas there were significantly less from P2 to P3 in the PG (*p* = 0.003). There were no significant (*p* = 0.89, *F* = 0.02, η_p_^2^ = 0.001) main effects of time, however, there was a group by time interaction (*p* = 0.002, *F* = 11.22, η_p_^2^ = 0.30) for changes in RDW. This variable was significantly less at P3 compared to P2 in PG (*p* = 0.016).

There were no significant (*p* = 0.08, *F* = 3.45, η_p_^2^ = 0.12) main effects of time, however, there was a group by time interaction (*p* = 0.004, *F* = 10.12, η_p_^2^ = 0.28) for changes in Hb. Post hoc showed that the Hb was significantly greater at P2 compared to P1, whilst, there were significant decreases at P3 compared to P2 for both groups. There were no significant (*p* = 0.96, *F* = 0.002, η_p_^2^ ≤ 0.001) main effects of time, however, there was a group by time interaction (*p* = 0.004, *F* = 9.99, η_p_^2^ = 0.28) for changes in Pts. Post hoc analysis showed a significant increase at P3 compared to P2 in PG (*p* = 0.001). There were no significant main effects of time for changes in Hct (*p* = 0.39, *F* = 0.76, η_p_^2^ = 0.03) or group by time interaction (*p* = 0.24, *F* = 1.45, η_p_^2^ = 0.05) (Table 3).

**Table 3:** Changes in blood cells counts levels between pre-post competitions season. Mean (M) ± standard deviation (SD)

Variables	Groups	Pre-Season	Mid-Season	Post-Season	Pre-Post Season		95% CI Hedge’s g	
		M ± SD	M ± SD	M ± SD	% Change	Hedge’s g	Lower	Upper
WBC (mm3)	*BG*	7321.4 ± 1544.8	6935.7 ± 1328.2	6528.6 ± 1583.3	−0.5	−1.2 L	0.3	−10.8
	*PG*	7220.0 ± 797.5	6966.7 ± 883.7	7540.0 ± 949.3^#^	0.3	−0.4 S	1.1	4.4
Neutrophils (%)	*BG*	53.3 ± 7.5	48.1 ± 3.9	41.1 ± 8.5	−1.4	−2.3 L	−0.6	−22.9
	*PG*	53.3 ± 8.2	57.6 ± 7.4	55.7 ± 14.0	0.2	−0.5 M	0.9	4.5
Lymphocytes (%)	*BG*	37.1 ± 7.0	42.6 ± 3.0	50.6 ± 7.3	1.8	0.9 L	2.7	36.4
	*PG*	37.6 ± 8.7	34.0 ± 7.0	35.9 ± 12.9	−0.1	−0.9 L	0.6	−4.5
NLR	*BG*	1.5 ± 0.5	1.1 ± 0.2	0.9 ± 0.3	−1.4	−2.3 L	−0.6	− 44.2
	*PG*	1.5 ± 0.6	1.8 ± 0.7	1.9 ± 1.1	0.4	−0.3 S	1.1	24.4
MIX (%)	*BG*	9.6 ± 1.7	9.3 ± 3.3	8.3 ± 3.5	−0.5	−1.2 L	0.3	−13.8
	*PG*	9.7 ± 2.1	8.9 ± 2.0	8.9 ± 2.6	−0.3	−1.0 L	0.4	−8.3
RBC (Mil.mm3)	*BG*	5.3 ± 0.3	5.2 ± 0.2	4.9 ± 0.5^*^	−1.1	−1.9 L	−0.3	−8.1
	*PG*	5.4 ± 0.4	5.4 ± 0.4	5.5 ± 0.3	0.3	−0.4 S	1.0	1.9
MCV (fL)	*BG*	81.5 ± 3.0	85.0 ± 2.8	84.2 ± 2.4	0.9	0.1 T	1.7	3.3
	*PG*	83.7 ± 4.0	86.3 ± 4.9	85.8 ± 5.6	0.4	−0.3 S	1.1	2.6
MCH (pg)	*BG*	27.9 ± 1.2	29.7 ± 1.0^€^	29.6 ± 1.0^*^	1.5	0.7 M	2.3	6.1
	*PG*	28.4 ± 1.9	29.7 ± 2.4^€^	29.2 ± 2.4^#*^	0.3	−0.4 S	1.0	2.5
MCHC (g/dL)	*BG*	34.3 ± 0.7	35.1 ± 1.0^€^	35.3 ± 1.1^*^	1.1	0.3 S	1.8	2.9
	*PG*	34.1 ± 1.4	34.6 ± 1.5	33.7 ± 2.1^#^	−0.2	−0.9 L	0.5	−1.1
RDW (%)	*BG*	13.5 ± 1.1	13.1 ± 0.8	13.3 ± 0.9	−0.3	−1.0 L	0.5	−2.0
	*PG*	13.2 ± 1.3	13.5 ± 1.1	13.1 ± 1.2^#^	−0.1	− 0.8 L	0.6	−1.1
Hb (g/dL)	*BG*	15.2 ± 1.0	15.9 ± 0.8^€^	15.6 ± 0.8^#^	0.4	−0.3 S	1.2	2.6
	*PG*	15.1 ± 1.3	15.9 ± 1.4^€^	15.1 ± 1.1^#^	0.0	−0.7 M	0.7	−0.1
Hct (%)	*BG*	44.4 ± 2.4	45.6 ± 2.4	45.5 ± 2.1	0.4	−0.3 S	1.2	2.4
	*PG*	44.7 ± 3.0	45.9 ± 2.8	45.0 ± 2.7	0.1	−0.6 M	0.8	0.8
Platelets (mm3)	*BG*	200,714.3 ± 22,269.1	192,357.1 ± 26,696.6	188,928.6 ± 36,389.9	−0.4	−1.1 L	0.4	−5.9
	*PG*	209,533.3 ± 43,843.7	209,200.0 ± 37,361.6	221,333.3 ± 36,112.3^#^	0.3	−0.4 S	1.0	5.6

*BG* Betaine Group, *PG* Placebo Group, *WBC* White blood cells, *MIX* Plural of eosinophils, basophils, and monocytes, *NLR* Neutrophils to lymphocytes ratio, *RBC* Red blood cells, *MCV* Mean corpuscular volume, *MCH* Mean corpuscular hemoglobin, *MCHC* Mean corpuscular hemoglobin concentration, *RDW* Red cell distribution width, *Hb* Hemoglobin, *Hct* Hematocrit; *P1* Pre-season, *P2* Mid-season, *P3* Post-season, *T* Trivial, *S* Small, *M* Moderate, *L* Large

^€^ significant difference compared to P1-P2 (*p* < 0.05);

^#^ significant difference compared to P2-P3 (*p* < 0.05);

^*^ significant difference compared to P1-P3 (*p* < 0.05)

Figure 1b-c shows the comparison of the mean difference between-group results with one-way ANOVA at the three assessments stages for CBC. WBC showed a difference in levels that were lower in the BG compared to the PG from P2 to P3 (*p* = 0.002) and P1 to P3 (*p* = 0.007). Neutrophils and NLR demonstrated that there was a difference in levels which were lower in the BG compared to PG from P1 to P2 (*p* ≤ 0.001) and P3 (*p* = 0.002). Lymphocytes demonstrated that there was a difference in levels which were greater compared to the BG with PG from P1 to P2 (*p* ≤ 0.001) and P3 (*p* = 0.001) (Fig. 1b). Pts levels decreased in P3 to P2 (*p* = 0.003) and P1 (*p* = 0.048) in BG compared to the PG. BG decreased in RDW levels in P2 to P1 (*p* = 0.014) compared to the PG, however, the PG decreased in P3 to P2 (*p* = 0.002) compared to the BG (Fig. 1b). BG decreased RBC levels in P3 to P2 (*p* = 0.019) and P1 (*p* = 0.003) compared to the PG. The reduction in Hb levels from P3 to P2 (*p* = 0.008) was less in the PG compared to the BG. MCH reduction levels in P3 to P2 (*p* = 0.019) was less in the BG compared to the PG, and increased levels in comparison at P3 to P1 (*p* = 0.007). MCHC demonstrated that there was a difference in levels which were greater compared to the BG with PG from P1 to P3 (*p* = 0.001) and P2 to P3 (*p* = 0.001) (Fig. 1c).

## Discussion

The aim of this study was to analyze the effect of betaine supplementation on the status of pro-inflammatory cytokines IL-1β, IL-6 and TNF-α, and the CBC counts in professional youth soccer players. We hypothesized that betaine supplementation would decrease the secretion of pro-inflammatory cytokines associated with a competitive soccer season. The results confirmed that 14 weeks of betaine supplementation prevented an increase in pro-inflammatory cytokines and WBC counts. These changes occurred despite both groups reporting a similar internal workload, consuming similar energy and macronutrients, and having similar levels of fatigue, stress, and sleep, as previously reported [13].

The competitive demands of soccer involve continuously performing high intensity eccentric actions, such as direction changes, accelerations, and decelerations, which, seems particularly damaging to the muscle [55–57]. When these demands are required chronically, fluctuations in hematological parameters occur [58, 59]. In the present study, inflammatory cytokines and WBCs were increased, and Hb, MCH, and MCHC were reduced at the end of the season in the PG. These changes in inflammatory markers are in line with another study that evaluated IL-6, at 5 different times during a season, in senior female soccer players and showed a linear increase [28]. These changes in basic indicators of pro-inflammatory status in athletes, when combined with changes in hematological parameters, point to a connection between NFOR and incomplete recovery and/or fatigue accumulation.

In the present study, inflammatory cytokines in the BG were 24–40% less at the end of the season compared to the start, whereas IL-1β (26%) and IL-6 (6.8%) were increased in the PG. IL-1β is produced in response to infection and injury, and its accumulation leads to intramuscular inflammation after eccentric exercise (17). Of the inflammatory cytokines assessed, IL-1β showed the greatest difference in response, decreasing by 40% between pre- and post-season in BG, but increasing by 26% in the PG. Betaine has been shown to suppress the NFκB pathway, and as a result, downregulates IL-1β and TNF-α gene expression and secretion [42]. The anti-inflammatory properties of betaine have been suggested to be beneficial for several diseases, including diabetes, cancer, and Alzheimer’s [39]. The changes in immune cell and inflammatory status in the present study suggest, for the first time, that betaine supplementation may also be a useful nutritional strategy to counter some of the negative immunological changes that are associated with NFOR.

Reductions in RBC, MCH, and Hb are the result of physical stress compounded over repeated competitions [60, 61], and have also been suggested to be indicative of the development of NFOR [27]. Previously published data from this ongoing study found an increase in testosterone and the testosterone to cortisol ratio during a competitive soccer season in this sample with betaine supplementation [13]. Testosterone has been shown to stimulate erythrocytosis [62] and cortisol increases Na (+), K (+)-ATPase activity to reduce erythrocyte volume [63]. Additionally, the addition of betaine to an in vitro medium in physiological concentrations has been shown to decrease hypoosmotic stress induced hemolysis by 42% via inhibition of erythrocyte membrane ATPases [64]. Given the aforementioned findings, we expected to see an improvement compared to placebo in anemia indicators over the course of the season. In the present study, betaine supplementation prevented a reduction in Hb, MCH, and RDW compared to placebo at the end of the season, but a decrease in RBC was observed in the BG. We are unable to speculate on what may account for these divergent results at this time. The improvement in Hb, MCH, and RDW suggests that betaine supplementation may protect erythrocytes against hemolysis during a soccer season, however, future research is necessary to further explore this hypothesis.

This study has limitations and proposals for future lines of research. First, although we instructed subjects to consume the same quantity and type of foods, we cannot discount that within subject differences in micronutrient intake may have influenced inflammatory and hematological parameters. Second, although we were able to track internal workload and indices of recovery, we did not have access to tools to measure external workloads, such as Global Positioning System devices [65–67]. Future research is necessary to investigate more closely the temporal changes in markers of inflammation over time with betaine supplementation, and to determine if the changes in inflammatory cytokines are sustainable with longer supplementation periods. Finally, additional research is necessary to measure changes in metrics of physical performance to better correlate the improvements in makers of inflammation, immune cells, and the testosterone to cortisol ratio [13] observed in response to betaine supplementation with NFOR.

## Conclusions

Fourteen weeks of betaine supplementation ameliorated an increase in IL-1β, IL-6, and TNF-α associated with the physical stressors of a competitive season in professional youth soccer players. These changes in pro-inflammatory cytokines and WBC suggest that betaine supplementation may be a useful nutritional strategy to regulate the immune system, and, together with the differences in Hb and MCH compared to PG, and the increase in the testosterone to cortisol ratio reported in this same sample [13], suggest that betaine supplementation may be used as part of a nutritional strategy to counter NFOR.

## Data Availability

The datasets used and/or analyzed during the current study are available from the corresponding author on reasonable request.

## Abbreviations

NFκB: nuclear factor kappa b pathway
IL-6: Interleukin-6
IL-1β: Interleukin-1 beta
TNF-α: Tumor necrosis factor-alpha
WBC: White blood cells
MIX: Plural of eosinophils, basophils, and monocytes
NLR: Neutrophils to lymphocytes ratio
RBC: Red blood cells
MCH: Mean corpuscular hemoglobin
MCHC: Mean corpuscular hemoglobin concentration
RDW: Red cell distribution width
Hb: Hemoglobin
Hct: Hematocrit
Plts: Platelets
NFOR: non-functional over reaching
ELISA: enzyme-like immune-sorbent assay
BG: betaine group
PG: placebo group
P1: pre-season
P2: mid-season
P3: post-season
